# *NCOA1* is a novel susceptibility gene for multiple myeloma in the Chinese population: A case-control study

**DOI:** 10.1371/journal.pone.0173298

**Published:** 2017-03-06

**Authors:** Mengle Peng, Guanfei Zhao, Funing Yang, Guixue Cheng, Jing Huang, Xiaosong Qin, Yong Liu, Qingtao Wang, Yongzhe Li, Dongchun Qin

**Affiliations:** 1 Department of Clinical Laboratory, The First Affiliated Hospital of Zhengzhou University, Key Laboratory of Laboratory Medicine of Henan Province, Zhengzhou, Henan, China; 2 Department of Clinical Laboratory, Beijing Chaoyang Hospital Affiliated to Capital Medical University, Beijing, China; 3 Department of Rheumatology and Clinical Immunology, Peking Union Medical College Hospital, Chinese Academy of Medical Sciences & Peking Union Medical College, Key Laboratory of Rheumatology and Clinical Immunology, Ministry of Education, Beijing, China; 4 Department of Clinical Laboratory, The First Hospital of Jilin University, Jilin, China; 5 Department of Clinical Laboratory, Shengjing Hospital Affiliated to China Medical University, Shenyang, Liaoning, China; Central South University, CHINA

## Abstract

Multiple myeloma (MM) is an incurable malignancy of mature B-lymphoid cells, and its pathogenesis is only partially understood. Previous studies have demonstrated that a number of Non-Hodgkin Lymphoma (NHL) associated genes also show susceptibility to MM, suggesting malignancies originating from B cells may share similar genetic susceptibility. Several recent large-scale genome-wide association studies (GWAS) have identified *HLA-I*, *HLA-II*, *CXCR5*, *ETS1*, *LPP* and *NCOA1* genes as genetic risk factors associated with NHL, and this study aimed to investigate whether these genes polymorphisms confer susceptibility with MM in the Chinese Han population. In 827 MM cases and 709 healthy controls of Chinese Han, seven single nucleotide polymorphisms (SNPs) in the *HLA–I* region (rs6457327), the *HLA–II* region (rs2647012 and rs7755224), the *CXCR5* gene (rs4938573), the *ETS1* gene (rs4937362), the *LPP* gene (rs6444305), and the *NCOA1* region (rs79480871) were genotyped using the Sequenom platform. Our study indicated that genotype and allele frequencies of rs79480871 showed strong associations with MM patients (*p*_*a*_ = 3.5×10^−4^ and *p*_*a*_ = 1.5×10^−4^), and the rs6457327 genotype was more readily associated with MM patients than with controls (*p*_*a*_ = 4.9×10^−3^). This study was the first to reveal the correlation between *NCOA1* gene polymorphisms and MM patients, indicating that *NCOA1* might be a novel susceptibility gene for MM patients in the Chinese Han population.

## Introduction

Multiple myeloma (MM) is the second most common hematological malignancy and is characterized by accumulation of clonal plasma B cells in bone marrow, hypercalcemia, renal failure, anemia, and lytic bone lesions. It has been estimated that every year, there are approximately 86,000 new invasive cases of MM, accounting for approximately 0.8% of all new cancer cases, and 63,000 related deaths, which represent 0.9% of all cancer death [1]. Incidence rates range from 0.4 to 5 per 100, 000, increasing markedly with age and with a male predominance [2]. Despite recent advances in the treatment of MM, the prognosis is poor and the genetic and molecular mechanisms underlying MM development remain unclear.

Recent genome-wide association studies (GWAS) have provided the first unambiguous evidence for genetic susceptibility to MM identifying single nucleotide polymorphisms (SNPs) affecting risk at chromosomes 2p33.3 (rs6746082), 3p22.1 (rs1052501), 3q26.2 (rs10936599), 5q15 (rs56219066T), 6p21.33(rs2285803), 7p15.3 (rs4487645), 11q13(rs603965), 17p11.2(rs4273077), and 22q13.1(rs877529) [3–6]. Among these chromosomes, rs603965 in the cyclin D1 (*CCND1*) gene at the exon 4 splice site was also associated with an increased risk of Non-Hodgkin Lymphoma (NHL) [7]. Furthermore, SNPs such as *CTLA-4*c.49 A>G (rs231775)[8] and *CASP9* Ex5 + 32 G>A (rs1052576) [9, 10] were also found to be associated with risk of MM and NHL, which support a genetic role for shared susceptibility that predisposes to these two B-cell origin malignancies. Until now, multiple GWAS of two common histological subtypes of NHL, follicular lymphoma (FL) and diffuse large B-cell lymphoma (DLBCL) have been performed, and several susceptibility loci have been identified, including human leukocyte antigen (*HLA*) class I and II regions (rs6457327, rs2647012 [11, 12] and rs7755224 [13]). Four susceptibility loci outside the *HLA* region have also been identified, including *CXCR5 (*rs4938573), *ETS1* (rs4937362), *LPP* (rs6444305) [14] and *NCOA1* (rs79480871) [15], which had strong associations with NHL. Hence, we chose these SNPs to determine if they confer susceptibility to MM. The human chromosome 6p21.3 region carries genes encoding major histocompatibility complex (MHC) proteins essential for the development of anti-infective and antitumor immune response and are the most polymorphic human genes [16]. Located in the *HLA* class 1 region at 6p21.33 near *HLA-C*, rs6457327 is inversely associated with risk of FL (*p* = 4.7×10^−11^) [17]. Rs2647012 and rs7755224 are located in the *MHC* Class II region at 6p21.32. Furthermore, rs2647012 has been demonstrated to be in close linkage disequilibrium (LD) with HLA-DRB1*15-DQA1*01-DQB1*06:02 haplotype, which is also associated with reduced risk of FL [18]. Rs2647012-linked variants significantly correlated with HLA expression change, particularly with increased *HLA-DQB1* gene expression [11]. Rs7755224 and rs10484561 are in complete LD (r^2^ = 1.0) and are located, respectively, 16 kb and 29 kb upstream of *HLA-DQB*. Evidence shows that rs7755224 in the *HLA* Class II region is strongly associated with FL susceptibility [13]. Rs4938573 at 11q23.3 maps 12.6 kb upstream of the chemokine (c-x-c motif) receptor 5 gene (*CXCR5*). The 11q24.3 locus marked by rs4937362 is approximately 35 kb upstream of v-ets avian erythroblastosis virus E26 oncogene homolog 1 (*ETS1*). The 3q28 locus marked by rs6444305 maps to a region that overlaps the LIM domain containing preferred translocation partner in lipoma (*LPP*) and is 836.4 kb upstream of *BCL6* [14]. The susceptibility locus at 2p23.3 (rs79480871) maps near *NCOA1*, nuclear receptor coactivator 1 and *ITSN2*, intersectin 2. *NCOA1* acts as a transcriptional coactivator for steroid and nuclear hormone receptors and is a member of the p160/steroid receptor coactivator (SRC) family 33 [15].

Considering the genetic overlap in B-cell origin malignancies and the associations of these genes with NHL, we hypothesized that the polymorphisms of rs6457327, rs2647012, rs7755224, rs4938573, rs4937362, rs6444305 and rs79480871 may be part of the genetic background that results in the development of MM in a Chinese Han population. Therefore, we developed the first large case-control study to determine the relationship between rs6457327, rs2647012, rs7755224, rs4938573, rs4937362, rs6444305 and rs79480871 polymorphisms and MM in a Chinese Han population.

## Methods

### Study population

The current study was designed as a case–control study and all subjects (MM, n = 827; control, n = 709) were unrelated and self-reported as Han Chinese ethnicity. In total, 827 MM patients and their clinical data were collected by the Department of Clinical Laboratory of The First Affiliated Hospital of Zhengzhou University (Zhengzhou, China), the Department of Clinical Laboratory of Beijing Chaoyang Hospital Affiliated to Capital Medical University (Beijing, China), the Rheumatology Department of Beijing Union Medical College Hospital (Beijing, China) and the Department of Clinical Laboratory of Shengjing Hospital Affiliated to China Medical University (Shenyang, China) between June 2015 and May 2016. MM was diagnosed according to standard criteria, which depends on the identification of abnormal monoclonal plasma cells in the bone marrow, M protein in the serum or urine, evidence of end-organ damage and a clinical picture consistent with MM [19]. MM was staged according to the Durie and Salmon staging system [20]. Patients’ characteristics at diagnosis, including age, gender, ISS stage, immunophenotype, the amplification of 1q21, p53 deletion, RB1 deletion, D13S319 deletion, hemoglobin (Hb), serum creatinine (Crea), serum albumin (Alb), β2-microglobulin (β2-MG) and serum calcium (Ca), were collected. Details regarding patients’ clinical and hematological features were reported in Table 1. In total, 709 ethnically matched healthy controls from these hospitals were recruited during their physical examinations according to the following rules: 1) at least 20 years old; 2) no personal history of lymphoma, leukemia, or HIV infection; and 3) no history of MM or known MGUS. This study was approved by the ethical committees of all participating centers. All participants signed a written informed consent form.

**Table 1:** The clinical characteristics of patients with MM enrolled in this study.

Characteristics	Number of patients n (%)	Number of controls n (%)
**Age,years (mean±SD)**	59.35±9.95	47.83±12.66
**Gender**		
Male	473 (57.2%)	409(57.7%)
Female	354 (42.8%)	300(42.3%)
***ISS***		
I	51 (6.2%)	
II	113 (13.7%)	
III	203 (24.5%)	
***Heavy chain paraprotein***		
IgG	353 (42.7%)	
IgA	161 (19.4%)	
IgD	24 (2.9%)	
IgM	4 (0.5%)	
LCO	194 (23.5%)	
None	31 (3.7%)	
NA/other	60 (7.3%)	
***Light chain paraprotein***		
Kappa	344 (41.6%)	
Lambda	389 (47.0%)	
None	31 (3.7%)	
NA/other	63 (7.7%)	
***Gain 1q21***		
YES	52 (6.3%)	
NO	35 (4.2%)	
NA	740 (89.5%)	
***Del p53***		
YES	45 (5.4%)	
NO	51 (6.2%)	
NA	731 (88.4%)	
***Del RB1***		
YES	62 (7.5%)	
NO	34 (4.1%)	
NA	731 (88.4%)	
***Del D13S319***		
YES	51 (6.1%)	
NO	39 (4.6%)	
NA	737 (89.3%)	
**Biochemical parameter (median; min-max)**		
Hb (g/L)	104.38 (29.8–169)	
Crea (umol/L)	119.15 (28–1194)	
Alb (g/L)	35.36 (11.4–64.5)	
β2-MG (mg/L)	5.99 (0.73–94.2)	
Ca (mmol/L)	2.18 (1.13–4.41)	

NA, not applicable; Hb: haemoglobin; Crea: creatinine; Alb: albumin; β2-MG: β2-microglobulin; Ca: calcium.

### Genotyping of selected SNPs

The DNA of all patients and controls were extracted from peripheral white blood cells with a genomic DNA kit (Tiangen, Beijing, China), following the manufacturer’s instructions. The DNA of each participant was genotyped using the Sequenom MassArray system (San Diego, CA, USA) according to the manufacturer’s protocol. Primers for the multiplex polymerase chain reaction (PCR) and for locus-specific single-base extension were designed by the MassArray Assay Design 4.0 software. The PCR was carried out in a 384 plate, and the products were used for locus-specific single-base extension reactions. The final products were then desalted and transferred to a 384-element SpectroCHIP array (Sequenom, CA). Allele detection was performed by matrix-assisted laser desorption ionization–time-of-flight mass spectrometry (MALDI-TOF MS). The resultant mass spectrogram data were analyzed using MassArray Typer software.

### Association analysis of the genotyped SNPs

The genotyped SNPs were tested for Hardy–Weinberg equilibrium (HWE) in the patient and control populations, and any SNPs that deviated from HWE (p < 0.05 in the control group) were excluded from subsequent analyses. Association analysis were analyzed using the PLINK tool set. Genotype and allele frequencies of the cases and controls were assessed using the χ^2^ test based on 2 × 3 and 2 × 2 contingency tables. Additionally, analysis under additive, dominant and recessive models were also performed. Furthermore, logistic regression analysis adjusting for age was performed. The odds ratio (OR) and 95% confidence interval (95% CI) were calculated, and *p* values (corrected for multiple testing by permutation (1,000,000 times)) less than 0.05 were considered statistically significant.

### Power analysis

We estimated the statistical power of this study using the Genetic Power Calculator program (http://pngu.mgh.harvard.edu/~purcell/gpc/cc2.html) and the following genetic model: a 12% risk allele frequency (similar to the minor allele frequency of rs79480871 T allele in our study); and a 0.001% prevalence rate of multiple myeloma in the Chinese population.

### Protein-protein Interaction network

To investigate the links between NCOA1 and well-defined MM susceptibility genes, a protein-protein Interaction (PPI) network based on InnateDB21 [21] was created using the NetworkAnalyst tools [22].

## Results

### Characteristics of participants

In this study, 827 MM patients (male/female, 473/354) and 709 ethnically and geographically matched healthy controls (male/female, 409/300) were collected from a Chinese Han population. Controls were sex-matched to the MM cases, and because MM is primarily diagnosed in patients over 60 years of age, the average age of cases was a little higher than that of our controls which were mainly recruited from young participants during their physical examinations. The fundamental characteristics of all the participators were illustrated in Table 1.

All seven polymorphisms were within Hardy-Weinberg equilibrium for the control group (*p* > 0.05) and the call rate > 95%. There was no deviation of the seven SNPs from HWE in the healthy controls. The genotyping success rates for rs6457327, rs2647012, rs7755224, rs4938573, rs4937362, rs6444305 and rs79480871 were 96.5%, 98.6%, 78.7%, 92.1%, 97.5%, 98.8% and 94.5%, respectively. Power analysis revealed a power of over 80% for detecting an association at a relative risk of 1.5–1.7 (for heterozygotes and homozygotes) using an additive model.

### Allele and genotype frequencies between cases and controls

Of the 7 nonsynonymous SNPs, six (rs6457327, rs2647012, rs4938573, rs4937362, rs6444305 and rs79480871) were successfully genotyped and one (rs7755224) failed (call rate < 80%). The distribution of both genotypic frequencies and allelic frequencies of the six SNPs is shown in Table 2. For the *HLA* class I region, the rs6457327 genotype was more readily associated with MM patients than with controls (*p*_*a*_ = 4.9×10^−3^), while for another SNP rs2647012 in the *HLA* class II region, no significant association was found with MM patients (*p*_*a*_ > 0.05). For the *CXCR5*, *ETS1* and *LPP* regions, none of these three SNPs (rs4938573, rs4937362 and rs6444305) demonstrated significant differences in allele or genotype frequencies between patients and controls (all, *p*_*a*_ > 0.05). For the *NCOA1* gene, the genotype and allele frequencies of rs79480871 manifested associations with MM patients (*p*_*a*_ = 3.5×10^−4^ and *p*_*a*_ = 1.5×10^−4^, OR: 1.67, 95% CI: 1.29–2.17, respectively).

**Table 2:** Allele and genotype distribution of the *HLA*, *CXCR5*, *ETS1*, *LPP* and *NCOA1* gene markers in MM patients and controls.

Gene	SNP	Genotypic test					Allelic test				
		Genotype	Case (n)/control (n)	*p*	*p*_*a*_	Χ^2^	Allele	Case (n)/control (n)	*p*	*p*_*a*_	OR (95% CI)
*HLA-I*	rs6457327	A/A	58/65	**3.8×10**^**−3**^	**4.9×10**^**−3**^	11.16	A	473/367	0.42	0.50	1.07 (0.91–1.26)
		A/C	357/237				C	1161/963			
		C/C	402/363								
*HLA-II*	rs2647012	T/T	65/38	0.18	0.23	3.47	T	425/325	0.15	0.19	1.13 (0.96–1.33)
		T/C	295/249				C	1223/1057			
		C/C	464/404								
*CXCR5*	rs4938573	C/C	17/14	0.79	1	0.46	C	195/146	0.61	0.62	1.06 (0.84–1.33)
		C/T	161/118				T	1387/1102			
		T/T	613/492								
*ETS1*	rs4937362	C/C	69/80	0.15	0.18	3.76	C	493/470	0.09	0.08	0.88 (0.75–1.02)
		C/T	355/310				T	1107/924			
		T/T	376/307								
*LPP*	rs6444305	G/G	21/21	0.49	0.67	1.44	G	300/243	0.53	0.86	1.06 (0.88–1.28)
		G/A	258/201				A	1340/1153			
		A/A	541/476								
*NCOA1*	rs79480871	T/T	16//6	**7.0×10**^**−4**^	**3.5×10**^**−4**^	14.53	T	188/93	**9.2×10**^**−5**^	**1.5×10**^**−4**^	1.67 (1.29–2.17)
		T/C	156/81				C	1434/1187			
		C/C	639/553								

CI, confidence interval; OR, odds ratio; *p*_*a*_, *p*-value corrected by permutation (1,000,000 times); SNP, single-nucleotide polymorphism.

Further analysis was performed based upon three genetic models (additive, dominant, and recessive models). The analysis outcomes of these three models are summarized in Table 3. Significant associations were observed in MM patients for rs79480871 in *NCOA1* gene region in the additive and dominant models (*p*_*a*_ < 0.05). For rs6457327 in the *HLA* class I region, an association was observed in the dominant model (*p*_*a*_ < 0.05). In the recessive model, a weak association was observed for rs4937362 in the *EST1* gene (*p*_*a*_ < 0.05). None of the three genetic models showed any significant differences between cases and controls for the three SNPs (rs2647012, rs4938573 and rs6444305) (all, *p*_*a*_ > 0.05, respectively).

**Table 3:** Analysis of the six SNPs based on three genetic models.

Gene	SNP	Additive model		Dominant model		Recessive model	
		*p*_*a*_	Χ^2^	*p*_*a*_	Χ^2^	*p*_*a*_	Χ^2^
*HLA-I*	rs6457327	0.75	0.65	**0.03**	4.25	0.07	3.45
*HLA-II*	rs2647012	0.15	2.01	0.44	0.71	0.06	3.38
*CXCR5*	rs4938573	0.67	0.24	1	0.37	1	0.01
*ETS1*	rs4937362	0.08	2.93	0.23	1.31	**0.048**	3.38
*LPP*	rs6444305	0.50	0.42	0.44	0.84	0.75	0.28
*NCOA1*	rs79480871	**1.1×10**^**−4**^	14.35	**2.1×10**^**−4**^	14.14	0.12	2.57

SNP, single-nucleotide polymorphism; *p*_*a*_, *p*-value corrected by permutation (1,000,000 times).

### Correlation between MM SNPs and the subphenotypes of MM

We also examined the associations between the SNPs and various clinical manifestations of MM. SNP rs4937362 of *EST1* demonstrated a correlation with heavy chain paraprotein (OR = 2.02, 95% CI: 1.18–3.48, *p* = 9.70×10^−3^) and light chain paraprotein (OR = 2.05, 95% CI: 1.16–3.63, *p* = 0.01), and the association still existed after permutation correction (*p*_*a*_ = 6.2×10^−3^ and *p*_*a*_ = 9.0×10^−3^). No association was found between rs6457327, rs2647012, rs4938573, rs6444305 and rs79480871 and any of the clinical features (*p* > 0.05) (Table 4).

**Table 4:** Association analysis of 6 SNPs with various clinical features.

Subphenotypes	Comparison	rs6457327		rs2647012		rs4938573		rs4937362		rs6444305		rs79480871	
		*p*	OR(95% CI)	*p*	OR(95% CI)	*p*	OR(95% CI)	*p*	OR(95% CI)	*p*	OR(95% CI)	*p*	OR(95% CI)
Heavy chain paraprotein	P (n = 542) vs N (n = 31)	0.75	1.08 (0.67–1.75)	0.26	1.35 (0.80–2.28)	0.16	1.76 (0.79–3.80)	**9.70×10**^**−3**^	2.02 (1.18–3.48)	0.34	1.36 (0.73–2.55)	0.85	1.07(0.54–2.11)
Light chain paraprotein	P (n = 194) vs N (n = 31)	0.83	1.06 (0.63–1.76)	0.44	1.25 (0.71–2.17)	0.44	1.39 (0.60–3.21)	**0.01**	2.05 (1.16–3.63)	0.14	1.64 (0.85–3.15)	0.82	0.92(0.44–1.91)
heavy vs. light	P (n = 542) vs P (n = 194)	0.86	0.98 (0.76–1.26)	0.56	0.92 (0.71–1.21)	0.21	0.79 (0.54–1.15)	0.91	1.11 (0.79–1.31)	0.22	1.20 (0.90–1.62)	0.43	0.86(0.59–1.25)
Gain 1q21	P (n = 52) vs N (n = 36)	0.52	1.23 (0.64–2.37)	0.60	1.21 (0.60–2.41)	0.65	1.24 (0.49–3.19)	0.17	1.60 (0.81–3.16)	0.07	0.40 (0.15–1.10)	0.55	1.40(0.46–4.30)
Del p53	P (n = 45) vs N (n = 51)	0.86	0.94 (0.50–1.79)	0.39	1.32 (0.70–2.48)	0.62	1.26 (0.51–3.12)	0.80	0.92 (0.49–1.73)	0.12	1.95 (0.83–4.54)	0.29	1.79(0.61–5.26)
Del RB1	P (n = 62) vs N (n = 34)	0.35	0.73 (0.38–1.41)	0.17	1.61 (0.82–3.15)	0.58	0.76 (0.29–2.00)	0.77	1.10 (0.57–2.11)	0.35	1.56 (0.62–3.91)	0.43	1.54(0.52–4.53)
Del D13S319	P (n = 51) vs N (n = 39)	0.63	0.85 (0.43–1.66)	0.10	1.74 (0.89–3.39)	0.33	0.60 (0.21–1.69)	0.70	0.88 (0.46–1.67)	0.22	1.74 (0.71–4.28)	0.17	2.11(0.72–6.21)
Low Hb levels	P (n = 454) vs N (n = 348)	0.32	0.90 (0.72–1.11)	0.19	1.16 (0.93–1.46)	0.6	0.92 (0.68–1.25)	0.35	1.11 (0.89–1.38)	0.82	0.97 (0.71–1.25)	0.90	0.98(0.72–1.34)
Low Alb levels	P (n = 341) vs N (n = 447)	0.87	1.02 (0.82–1.27)	0.18	0.85 (0.68–1.08)	0.75	1.05 (0.78–1.43)	0.35	1.11 (0.89–1.38)	0.69	1.05 (0.81–1.37)	0.28	0.84(0.61–1.15)
High Crea levels	P (n = 161) vs N (n = 627)	0.32	0.87 (0.66–1.15)	0.76	0.96 (0.72–1.27)	0.26	0.79 (0.53–1.19)	0.94	1.01 (0.77–1.33)	0.38	0.86 (0.62–1.20)	0.82	0.96(0.65–1.41)
High β2-MG levels	P (n = 452) vs N (n = 172)	0.37	0.88 (0.68–1.16)	0.48	0.90 (0.68–1.20)	0.65	0.65 (0.63–1.33)	0.79	0.96 (0.73–1.27)	0.17	1.26 (0.91–1.75)	0.72	1.07(0.72–1.59)

P: patients positive for a certain phenotype; N: patients negative for a certain phenotype; C: controls. Hb: haemoglobin; Alb: albumin; Crea: creatinine; β2-MG: β2-microglobulin; Del, deletion; permutation corrections data not shown.

## Discussion

To our knowledge, the current study represents the largest genetic association study performed in MM to date and the first to test the association of rs6457327, rs2647012, rs7755224, rs4938573, rs4937362, rs6444305 and rs79480871 polymorphisms with MM in a Chinese Han population. We chose to investigate the genetic contribution of rs6457327, rs2647012, rs7755224, rs4938573, rs4937362, rs6444305 and rs79480871 to MM not only because the strong associations reported for NHL, but also based upon the putative roles that the two B-cell origin malignancies (MM and NHL) may share in common genetic susceptibility. The current study performed the first genetic analysis to associate *NCOA1* with the pathogenesis of MM in a Han Chinese population. Our study confirmed that Chinese Han patients carrying the *NCOA1* rs79480871-T allele were at increased risk for developing MM. In addition, associations were found between rs79480871 and MM under additive model and dominant model, and we suggested that rs79480871 was a putative susceptible gene for MM in Chinese Han patients. Together, these results support the notion that rs79480871 acts as a common genetic factor in the pathogenesis of MM and NHL.

The susceptibility locus at 2p23.3 (rs79480871) maps near *NCOA1*, nuclear receptor coactivator 1 and *ITSN2*, intersectin 2 [15]. *NCOA1* acts as a transcriptional coactivator for steroid and nuclear hormone receptors and is a member of the p160/ SRC family 33 that also includes *NCOA2* and *NCOA3*. These SRC coactivators not only play pivotal roles in development, growth, reproduction and metabolism, but also play crucial roles in cancer [23]. *NCOA1* is overexpressed in 19–29% of human breast tumors and its overexpression positively correlates with HER2 expression, lymph node metastasis, disease recurrence and poor survival [24–26]. Recently, a study reported that *NCOA1* worked with hypoxia-inducible factor-1α *(HIF1α)* and AP-1 (c-Jun/c-Fos) to promote vascular endothelial growth factor (*VEGF*, also termed *VEGFa*) expression in breast cancer cells and drove breast tumor angiogenesis in both mouse and human breast tumors, which suggested that *NCOA1*-promoted breast cancer metastasis might be related to its role in angiogenesis [27]. In addition, high *NCOA1* expression concomitant with high micro-vessel density (MVD) in breast tumors has been associated with poor prognosis. Interestingly, *HIF1α* has been regarded as the most important transcriptional factor promoting angiogenesis by upregulating pro-angiogenic genes such as *VEGF*, which can enhance the MVD of bone marrow and accounts for the abnormal structure of myeloma tumor vessels [28, 29]. Therefore, we inferred that *NCOA1* might serve as a new molecular target for inhibiting MM angiogenesis and metastasis through HIF1α and AP-1-mediated *VEGFa* transcription. Furthermore, the current study only identified *NCOA1* (rs79480871) susceptibility to MM, which was found in GWAS of DLBCL, while it is also necessary to perform fine mapping analysis of the whole gene.

To gain further insight into the potential relationships between *NCOA1* and well-defined MM susceptibility genes (S1 Table), we constructed PPI networks from the literature-curated human interactome. Interestingly, among the protein-protein interactions, *NCOA1* was identified as a major hub node with the sixth highest degree at 77, implying that *NCOA1* has connections with many other MM susceptibility genes nodes, and should be a novel member involved in the biological processes underlying MM susceptibility (S1 Fig). *NCOA1* was identified to directly interact with vitamin D receptor (*VDR*) gene, which encodes a nuclear transcription-regulating factor that drives the synthesis of proteins involved in bone mineral homeostasis and cell cycle regulation [30], and the FokI polymorphism (rs2228570) of *VDR* has been involved in the increased susceptibility to development and progression in multiple myeloma in the ethnic Kashmiri population [31]. Moreover, the biological connection between *NCOA1* and MM susceptibility genes may help in gaining deeper insights into the underlying disease mechanisms and revealing more intricate biological processes associated with disease development.

*HLA* class I- and class II-restricted CD8+ and CD4+ T-cell responses are essential for the immune system to mount a successful anti-tumor immune defense or to remove infected [18]. In a previous study, the HLA-A*03 and HLA-B*18 alleles were shown to have significant susceptibility effects on MM in the Iranian population [32]. The 2 *HLA* class I/II SNPs (rs6457327, rs2647012) were identified to date as susceptibility loci for NHL subtypes and have largely been associated with FL [33, 34]. Wrench et al suggest that the SNP rs6457327 is a predictive marker for the transformation of FL to DLBCL [35]. In addition, FL patients who later transform to DLBCL have a significantly worse prognosis if they carry the AA or AC genotype compared with patients carrying the CC genotype at SNP rs6457327 [36]. In the current study, the AA genotype frequencies of rs6457327 may in fact play a dominant role in the pathogenesis of MM, but no association was found between MM and rs2647012. Five non-*HLA* loci that achieved genome-wide significance (*p* < 5×10^−8^) at 11q23.3 (rs4938573, *p* < 5.79×10^−20^), 11q24.3 (rs4937362, *p* < 6.76×10^−11^), 3q28 (rs6444305, *p* < 1.10×10^−10^), 18q21.33 (rs17749561, *p* < 8.28×10^−10^), and 8q24.21 (rs13254990, *p* < 5×10^−8^) for FL were identified by Skibola et al [14]. Three of them were tested in our study, and we failed to demonstrate an association between *CXCR5* (rs4938573) and *LPP* (rs6444305) and the risk of MM patients. Only *EST1* (rs4937362) showed a weak association in the recessive model.

In addition, we demonstrate a correlation between rs4937362 of *EST1* with heavy chain paraprotein and light chain paraprotein, and the association still existed after permutation correction. However, our study failed to analyze the potential association of these genetic variants with other clinical subtypes of MM in this population. This failure may be attributed to the insufficient sample size of subtypes leading to a failure to detect potential associations. Next, because the age of controls was lower than that of cases, logistic regression analysis adjusting for age was performed to decrease potential confounding factors and biases; the results corrected by permutation (1,000,000 times) showed that rs79480871 remains remarkably significant (*p*_*a*_ < 0.05), indicating the stability of our results (S2 Table). Lastly, our study was limited at the genetic level, and thus functional studies will be required to elucidate the biological basis of these loci and to determine their role in MM.

In summary, the current study was the largest genetic association study performed in MM in the Chinese Han population to date, and the first investigation to indicate that the *NCOA1* region (rs79480871) might be the susceptibility gene for MM patients. Future studies on MM patients using larger sample sizes should be performed to confirm these outcomes. In addition, a larger sample size and more SNPs might be required for further analysis of the association between *NCOA1* and MM susceptibility in different ethnic populations.

## Supporting information

S1 Table Genes and the relevant SNPs involved in risk of MM. (DOCX)

S2 Table Logistic regression analysis adjusting for age of the *HLA*, *CXCR5*, *ETS1*, *LPP* and *NCOA1* gene markers in MM patients and controls. OR, odds ratio; CI, confidence interval; *p*_*a*_, *p*-value corrected by permutation (1,000,000 times); SNP, single-nucleotide polymorphism.
(DOC)

S1 Fig Protein-protein Interaction (PPI) network. The top hub nodes of the network were shown by the red circle.
(TIF)

S1 Data SNP genotying data of the study (PDF). (PDF)
