# Supplementary material for: NCOA1 is a novel susceptibility gene for multiple myeloma in the Chinese population: A case-control study
Source: PLoS One. 2017 Mar 6;12(3):e0173298. doi: 10.1371/journal.pone.0173298 (PMC5338790; doi:10.1371/journal.pone.0173298)
Supplement: S1 Table — (DOCX) [file pone.0173298.s001.docx]

**S1 Table.** Genes and the relevant SNPs involved in risk of MM.

| Genes | SNPs | references |
| --- | --- | --- |
| ULK4 | rs1052501 | [1] |
| DNAH11 | rs487645 | [1] |
| DTNB | rs6746082 | [1] |
| CCND1 | rs603965 | [2] |
| MYMN | rs10936599 | [3] |
| PSORS1C2 | rs2285803 | [3] |
| TNFRSF13B | rs4273077 | [3] |
| CBX7 | rs877529 | [3] |
| ST6GAL2 | rs12614346 | [4] |
| ELL2 | rs56219066 | [5] |
| CTLA4 | rs231775,rs3087243,rs11571302 | [6] |
| BAX | rs1042265 | [7] |
| RIPK1 | rs9391981 | [7] |
| CASP3 | rs1049216 | [8] |
| CASP9 | rs1052576, rs751643 | [8] |
| SERPINE1 | rs2227667 | [9] |
| HGF | rs17501108 | [9] |
| IL1A | rs1800587 | [10] |
| IL1B | rs1143627 | [11] |
| IL-6 | rs1800795 | [12] |
| IL-6R | rs6684439,rs75729229,rs2228145 | [13] |
| TNF-α | rs1800629,rs361525 | [14] |
| IGF1 | rs7965399,rs2195239,rs2373722 | [13] |
| HPSE | rs4693602 | [7] |
| IRS1 | rs1801278,rs17208470 | [13] |
| MDR1 | rs1045642 | [15] |
| MTHFR | rs1801133,rs1801131 | [16] |
| XRCC4 | rs963248 | [17] |
| XRCC5 | rs1051685 | [17] |
| CYP1A1 | rs4646903 | [18] |
| CYP1B1 | rs1056836 | [19] |
| VEGF | rs699947,rs1570360,rs2010963 | [20] |
| VEGF2 | rs2071559,rs2305948 | [20] |
| VDR | rs2228570 | [21] |

**References**

[1]. Broderick P, Chubb D, Johnson DC, Weinhold N, Forsti A, Lloyd A, et al. Common variation at 3p22.1 and 7p15.3 influences multiple myeloma risk. Nature genetics. 2011;44(1):58-61. Epub 2011/11/29. doi: 10.1038/ng.993. PubMed PMID: 22120009; PubMed Central PMCID: PMCPmc5108406.

[2]. Weinhold N, Johnson DC, Chubb D, Chen B, Forsti A, Hosking FJ, et al. The CCND1 c.870G>A polymorphism is a risk factor for t(11;14)(q13;q32) multiple myeloma. Nature genetics. 2013;45(5):522-5. Epub 2013/03/19. doi: 10.1038/ng.2583. PubMed PMID: 23502783; PubMed Central PMCID: PMCPmc5056630.

[3]. Chubb D, Weinhold N, Broderick P, Chen B, Johnson DC. Common variation at 3q26.2, 6p21.33, 17p11.2 and 22q13.1 influences multiple myeloma risk. 2013;45(10):1221-5. doi: 10.1038/ng.2733. PubMed PMID: 23955597.

[4]. Erickson SW, Raj VR, Stephens OW, Dhakal I, Chavan SS, Sanathkumar N, et al. Genome-wide scan identifies variant in 2q12.3 associated with risk for multiple myeloma. Blood. 2014;124(12):2001-3. Epub 2014/09/23. doi: 10.1182/blood-2014-07-586701. PubMed PMID: 25237182.

[5]. Swaminathan B, Thorleifsson G, Joud M, Ali M, Johnsson E, Ajore R, et al. Variants in ELL2 influencing immunoglobulin levels associate with multiple myeloma. 2015;6:7213. doi: 10.1038/ncomms8213. PubMed PMID: 26007630.

[6]. Karabon L, Pawlak-Adamska E, Tomkiewicz A, Jedynak A, Kielbinski M, Woszczyk D, et al. Variations in suppressor molecule ctla-4 gene are related to susceptibility to multiple myeloma in a polish population. Pathology oncology research : POR. 2012;18(2):219-26. Epub 2011/07/12. doi: 10.1007/s12253-011-9431-6. PubMed PMID: 21744007; PubMed Central PMCID: PMCPmc3313022.

[7]. Hosgood HD, 3rd, Baris D, Zhang Y, Berndt SI, Menashe I, Morton LM, et al. Genetic variation in cell cycle and apoptosis related genes and multiple myeloma risk. Leukemia research. 2009;33(12):1609-14. Epub 2009/04/14. doi: 10.1016/j.leukres.2009.03.013. PubMed PMID: 19362737; PubMed Central PMCID: PMCPmc2749910.

[8]. Hosgood HD, 3rd, Baris D, Zhang Y, Zhu Y, Zheng T, Yeager M, et al. Caspase polymorphisms and genetic susceptibility to multiple myeloma. Hematological oncology. 2008;26(3):148-51. Epub 2008/04/03. doi: 10.1002/hon.852. PubMed PMID: 18381704; PubMed Central PMCID: PMCPmc2586415.

[9]. Purdue MP, Lan Q, Menashe I, Zheng T, Zhang Y, Yeager M, et al. Variation in innate immunity genes and risk of multiple myeloma. Hematological oncology. 2011;29(1):42-6. Epub 2010/07/27. doi: 10.1002/hon.954. PubMed PMID: 20658475; PubMed Central PMCID: PMCPmc2980579.

[10]. Abazis-Stamboulieh D, Oikonomou P, Papadoulis N, Panayiotidis P, Vrakidou E, Tsezou A. Association of interleukin-1A, interleukin-1B and interleukin-1 receptor antagonist gene polymorphisms with multiple myeloma. Leukemia & lymphoma. 2007;48(11):2196-203. Epub 2007/10/11. doi: 10.1080/10428190701615892. PubMed PMID: 17926179.

[11]. Vangsted AJ, Klausen TW, Ruminski W, Gimsing P, Andersen NF, Gang AO, et al. The polymorphism IL-1beta T-31C is associated with a longer overall survival in patients with multiple myeloma undergoing auto-SCT. Bone marrow transplantation. 2009;43(7):539-45. Epub 2008/11/11. doi: 10.1038/bmt.2008.351. PubMed PMID: 18997828.

[12]. Cozen W, Gebregziabher M, Conti DV, Van Den Berg DJ, Coetzee GA, Wang SS, et al. Interleukin-6-related genotypes, body mass index, and risk of multiple myeloma and plasmacytoma. Cancer epidemiology, biomarkers & prevention : a publication of the American Association for Cancer Research, cosponsored by the American Society of Preventive Oncology. 2006;15(11):2285-91. Epub 2006/11/23. doi: 10.1158/1055-9965.epi-06-0446. PubMed PMID: 17119059.

[13]. Birmann BM, Tamimi RM, Giovannucci E, Rosner B, Hunter DJ, Kraft P, et al. Insulin-like growth factor-1- and interleukin-6-related gene variation and risk of multiple myeloma. Cancer epidemiology, biomarkers & prevention : a publication of the American Association for Cancer Research, cosponsored by the American Society of Preventive Oncology. 2009;18(1):282-8. Epub 2009/01/07. doi: 10.1158/1055-9965.epi-08-0778. PubMed PMID: 19124510; PubMed Central PMCID: PMCPmc2661109.

[14]. Du J, Yuan Z, Zhang C, Fu W, Jiang H, Chen B, et al. Role of the TNF-alpha promoter polymorphisms for development of multiple myeloma and clinical outcome in thalidomide plus dexamethasone. Leukemia research. 2010;34(11):1453-8. Epub 2010/02/04. doi: 10.1016/j.leukres.2010.01.011. PubMed PMID: 20122728.

[15]. Yin G, Xiao Z, Ni Y, Qu X, Wu H, Lu H, et al. Association of MDR1 single-nucleotide polymorphisms and haplotype variants with multiple myeloma in Chinese Jiangsu Han population. Tumour biology : the journal of the International Society for Oncodevelopmental Biology and Medicine. 2016;37(7):9549-54. Epub 2016/01/23. doi: 10.1007/s13277-015-4574-9. PubMed PMID: 26790444.

[16]. Ma LM, Ruan LH, Yang HP. Meta-analysis of the association of MTHFR polymorphisms with multiple myeloma risk. Scientific reports. 2015;5:10735. Epub 2015/05/30. doi: 10.1038/srep10735. PubMed PMID: 26022785; PubMed Central PMCID: PMCPmc4448268.

[17]. Hayden PJ, Tewari P, Morris DW, Staines A, Crowley D, Nieters A, et al. Variation in DNA repair genes XRCC3, XRCC4, XRCC5 and susceptibility to myeloma. Human molecular genetics. 2007;16(24):3117-27. Epub 2007/09/29. doi: 10.1093/hmg/ddm273. PubMed PMID: 17901044.

[18]. Kang SH, Kim TY, Kim HY, Yoon JH, Cho HI, Yoon SS, et al. Protective role of CYP1A1*2A in the development of multiple myeloma. Acta haematologica. 2008;119(1):60-4. Epub 2008/02/21. doi: 10.1159/000117572. PubMed PMID: 18285692.

[19]. Gold LS, De Roos AJ, Brown EE, Lan Q, Milliken K, Davis S, et al. Associations of common variants in genes involved in metabolism and response to exogenous chemicals with risk of multiple myeloma. Cancer epidemiology. 2009;33(3-4):276-80. Epub 2009/09/09. doi: 10.1016/j.canep.2009.08.005. PubMed PMID: 19736056; PubMed Central PMCID: PMCPmc2808169.

[20]. Brito AB, Lourenco GJ, Oliveira GB, De Souza CA, Vassallo J, Lima CS. Associations of VEGF and VEGFR2 polymorphisms with increased risk and aggressiveness of multiple myeloma. Annals of hematology. 2014;93(8):1363-9. Epub 2014/04/02. doi: 10.1007/s00277-014-2062-8. PubMed PMID: 24687381.

[21]. Shafia S, Qasim I, Aziz SA, Bhat IA, Nisar S, Shah ZA. Role of vitamin D receptor (VDR) polymorphisms in susceptibility to multiple myeloma in ethnic Kashmiri population. Blood cells, molecules & diseases. 2013;51(1):56-60. Epub 2013/03/05. doi: 10.1016/j.bcmd.2013.02.001. PubMed PMID: 23453529.
