# Supplementary material for: NCOA1 is a novel susceptibility gene for multiple myeloma in the Chinese population: A case-control study
Source: PLoS One. 2017 Mar 6;12(3):e0173298. doi: 10.1371/journal.pone.0173298 (PMC5338790; doi:10.1371/journal.pone.0173298)
Supplement: S2 Table — OR, odds ratio; CI, confidence interval; pa, p-value corrected by permutation (1,000,000 times); SNP, single-nucleotide polymorphism. (DOC) [file pone.0173298.s002.doc]

| **Gene** | **SNP** | **Allele** | **OR** | **95% CI** | ***p*** | ***p_a_*** |
| --- | --- | --- | --- | --- | --- | --- |
| *HLA-I* | rs6457327 | A | 1.052 | 0.879-1.259 | 0.581 | 0.750 |
| *HLA-II* | rs2647012 | T | 1.073 | 0.893-1.288 | 0.451 | 0.562 |
| *CXCR5* | rs4938573 | C | 0.999 | 0.780-1.280 | 0.995 | 1 |
| *ETS1* | rs4937362 | C | 0.876 | 0.736-1.043 | 0.138 | 0.206 |
| *LPP* | rs6444305 | G | 1.154 | 0.932-1.430 | 0.189 | 0.214 |
| *NCOA1* | rs79480871 | T | 1.577 | 1.185-2.097 | **1.76×10^-3^** | **1.82×10^-3^** |

**S2 Table. Logistic regression analysis adjusting for age of the *HLA*, *CXCR5*, *ETS1*, *LPP* and *NCOA1* gene markers in MM patients and controls.**

OR, odds ratio; CI, confidence interval; *p_a_*, *p*-value corrected by permutation (1,000,000 times); SNP, single-nucleotide polymorphism.
