# Supplementary figures and images for: NCOA1 is a novel susceptibility gene for multiple myeloma in the Chinese population: A case-control study
Source: PLoS One. 2017 Mar 6;12(3):e0173298. doi: 10.1371/journal.pone.0173298 (PMC5338790; doi:10.1371/journal.pone.0173298)

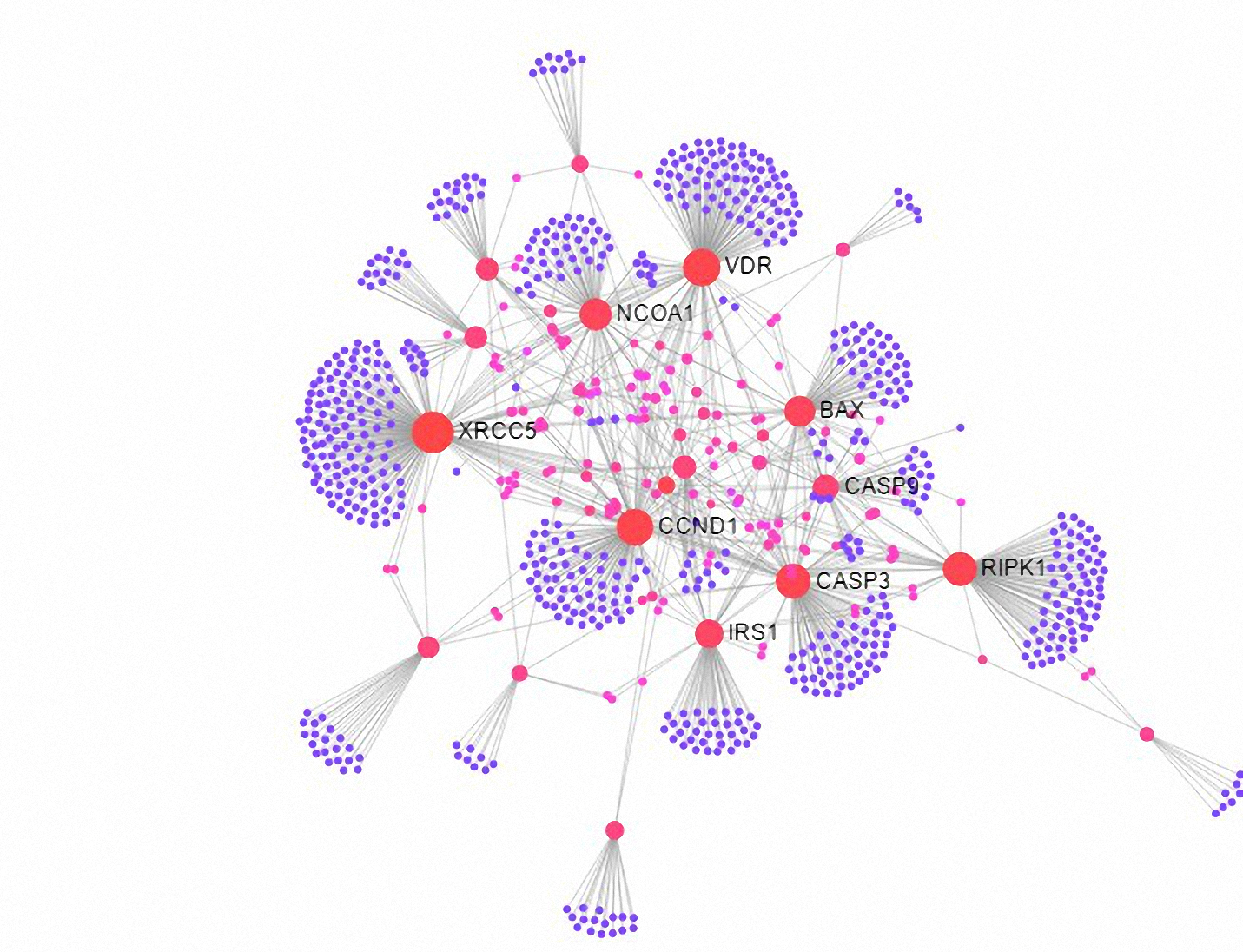

Supplement: S1 Fig — The top hub nodes of the network were shown by the red circle. (TIF) [file pone.0173298.s003.tif]
