# Supplementary material for: NCOA1 is a novel susceptibility gene for multiple myeloma in the Chinese population: A case-control study
Source: PLoS One. 2017 Mar 6;12(3):e0173298. doi: 10.1371/journal.pone.0173298 (PMC5338790; doi:10.1371/journal.pone.0173298)
Supplement: S1 Data — (PDF) [file pone.0173298.s004.pdf]

Number	Gender	Age	Type	rs2647012	rs4938573	rs6444305	rs6457327	rs79480871	rs4937362
1	Male	62	multiple myeloma	C/C	0/0	A/A	C/A	C/T	0/0
2	Male	49	multiple myeloma	C/C	T/T	A/A	C/A	C/C	0/0
3	Feale	57	multiple myeloma	C/C	T/T	G/A	C/C	C/C	C/T
4	Male	59	multiple myeloma	T/C	T/T	A/A	C/A	C/C	C/T
5	Feale	63	multiple myeloma	C/C	T/T	A/A	C/C	C/C	C/C
6	Feale	54	multiple myeloma	T/C	T/T	A/A	C/A	C/C	T/T
7	Male	86	multiple myeloma	T/C	C/C	A/A	C/A	C/C	T/T
8	Male	52	multiple myeloma	C/C	T/T	A/A	C/A	C/C	0/0
9	Male	56	multiple myeloma	C/C	T/T	G/A	C/A	C/C	C/T
10	Feale	67	multiple myeloma	T/C	T/T	A/A	C/C	C/C	0/0
11	Male	55	multiple myeloma	T/C	T/T	A/A	C/C	C/C	0/0
12	Male	60	multiple myeloma	T/C	C/T	A/A	C/A	C/C	T/T
13	Male	51	multiple myeloma	C/C	T/T	A/A	C/A	0/0	T/T
14	Feale	59	multiple myeloma	C/C	C/C	G/A	A/A	C/C	C/T
15	Male	53	multiple myeloma	C/C	T/T	A/A	C/C	C/C	C/C
16	Male	58	multiple myeloma	T/C	T/T	A/A	C/A	C/C	C/T
17	Male	46	multiple myeloma	C/C	C/T	A/A	C/C	C/T	C/T
18	Male	52	multiple myeloma	T/C	T/T	G/A	C/C	C/C	C/C
19	Feale	48	multiple myeloma	T/C	C/T	A/A	C/C	C/C	C/T
20	Feale	58	multiple myeloma	T/C	0/0	A/A	C/C	C/C	T/T
21	Male	57	multiple myeloma	T/C	T/T	G/A	C/C	C/C	T/T
22	Male	61	multiple myeloma	C/C	T/T	A/A	A/A	C/C	T/T
23	Feale	47	multiple myeloma	C/C	T/T	G/A	C/A	C/T	0/0
24	Male	54	multiple myeloma	T/T	C/T	A/A	C/A	C/C	C/T
25	Feale	62	multiple myeloma	T/C	T/T	G/G	C/A	C/T	T/T
26	Male	66	multiple myeloma	T/C	T/T	A/A	C/C	C/C	C/T
27	Feale	45	multiple myeloma	T/C	C/T	A/A	A/A	C/C	0/0
28	Male	59	multiple myeloma	T/C	T/T	G/A	C/C	C/C	C/T
29	Male	59	multiple myeloma	C/C	T/T	A/A	C/A	C/C	T/T
30	Feale	56	multiple myeloma	T/C	T/T	A/A	C/A	C/C	T/T
31	Male	62	multiple myeloma	T/C	T/T	A/A	C/C	C/C	0/0
32	Male	50	multiple myeloma	C/C	0/0	G/A	C/C	C/C	C/T
33	Male	52	multiple myeloma	C/C	C/T	A/A	C/C	C/C	C/T
34	Male	50	multiple myeloma	T/C	T/T	A/A	C/C	C/C	0/0
35	Male	51	multiple myeloma	C/C	T/T	A/A	C/A	C/C	C/T
36	Male	74	multiple myeloma	C/C	T/T	A/A	C/A	C/C	T/T
37	Male	49	multiple myeloma	C/C	T/T	A/A	C/A	C/C	C/T
38	Feale	50	multiple myeloma	T/C	T/T	A/A	C/A	C/C	C/T
39	Male	52	multiple myeloma	C/C	T/T	A/A	C/C	C/T	C/T
40	Male	71	multiple myeloma	C/C	C/T	A/A	C/A	C/T	C/T
41	Feale	49	multiple myeloma	C/C	T/T	G/A	C/A	C/T	T/T
42	Male	61	multiple myeloma	T/C	T/T	A/A	C/C	C/C	C/C
43	Feale	66	multiple myeloma	C/C	T/T	A/A	A/A	C/C	C/T
44	Male	73	multiple myeloma	T/C	T/T	A/A	C/A	C/T	T/T
45	Feale	49	multiple myeloma	T/C	T/T	A/A	C/A	C/C	C/T
46	Feale	47	multiple myeloma	C/C	C/T	A/A	C/A	C/C	T/T
47	Male	67	multiple myeloma	C/C	T/T	A/A	C/C	C/C	T/T
48	Feale	20	multiple myeloma	T/C	T/T	G/A	C/C	C/C	C/T
49	Male	70	multiple myeloma	C/C	C/T	A/A	C/A	C/T	C/T
50	Male	43	multiple myeloma	C/C	T/T	A/A	C/A	C/C	C/T
51	Male	47	multiple myeloma	C/C	T/T	G/A	C/A	C/T	C/T
52	Male	59	multiple myeloma	C/C	T/T	A/A	C/A	C/C	T/T
53	Male	47	multiple myeloma	C/C	T/T	A/A	C/C	C/C	C/T
54	Male	64	multiple myeloma	C/C	T/T	A/A	C/C	C/T	T/T
55	Feale	57	multiple myeloma	T/C	T/T	G/A	C/A	C/C	C/T
56	Male	63	multiple myeloma	T/C	T/T	G/A	C/C	C/C	C/C
57	Male	63	multiple myeloma	C/C	T/T	G/A	C/C	C/T	C/T
58	Male	67	multiple myeloma	T/C	C/T	A/A	C/A	C/T	T/T
59	Feale	59	multiple myeloma	T/T	T/T	A/A	C/C	C/C	T/T
60	Male	49	multiple myeloma	T/C	T/T	A/A	C/A	C/C	T/T
61	Feale	44	multiple myeloma	T/T	T/T	A/A	C/A	C/C	T/T
62	Feale	36	multiple myeloma	C/C	T/T	G/A	C/A	C/C	T/T
63	Male	73	multiple myeloma	T/C	T/T	A/A	C/A	C/C	C/T
64	Feale	62	multiple myeloma	T/C	T/T	A/A	C/A	C/C	C/T
65	Male	70	multiple myeloma	T/C	T/T	A/A	C/C	C/C	C/T
66	Feale	51	multiple myeloma	T/C	T/T	G/A	C/C	C/C	C/T
67	Male	56	multiple myeloma	C/C	T/T	A/A	C/A	C/C	T/T
68	Feale	51	multiple myeloma	T/T	C/T	A/A	C/C	C/C	C/T
69	Male	68	multiple myeloma	T/C	T/T	G/A	C/C	C/C	C/T
70	Male	58	multiple myeloma	C/C	T/T	A/A	C/A	C/C	C/T
71	Male	53	multiple myeloma	T/C	T/T	A/A	C/A	C/C	T/T
72	Male	63	multiple myeloma	T/T	C/C	G/A	A/A	C/C	T/T
73	Feale	65	multiple myeloma	C/C	T/T	G/A	C/C	C/C	C/C
74	Feale	58	multiple myeloma	C/C	T/T	A/A	A/A	C/C	T/T
75	Feale	59	multiple myeloma	T/C	C/T	A/A	C/A	C/C	T/T
76	Male	60	multiple myeloma	T/C	T/T	A/A	C/C	C/C	T/T
77	Male	68	multiple myeloma	T/C	T/T	A/A	C/C	C/C	T/T
78	Feale	62	multiple myeloma	T/C	T/T	A/A	C/A	C/C	C/T
79	Male	57	multiple myeloma	T/C	T/T	A/A	C/A	C/T	0/0
80	Male	62	multiple myeloma	T/C	C/T	G/A	C/C	C/C	C/T
81	Feale	66	multiple myeloma	C/C	T/T	A/A	C/A	C/T	T/T
82	Feale	61	multiple myeloma	C/C	T/T	A/A	C/A	C/C	C/T
83	Male	75	multiple myeloma	C/C	T/T	G/A	A/A	C/T	T/T
84	Male	45	multiple myeloma	T/C	T/T	A/A	C/A	C/C	C/T
85	Feale	60	multiple myeloma	C/C	T/T	G/A	C/A	C/C	T/T
86	Feale	64	multiple myeloma	T/C	T/T	A/A	C/C	T/T	T/T
87	Feale	48	multiple myeloma	T/C	T/T	A/A	C/A	C/T	T/T
88	Male	66	multiple myeloma	C/C	T/T	A/A	C/C	C/C	0/0
89	Male	61	multiple myeloma	T/T	0/0	G/A	C/C	C/C	T/T
90	Feale	68	multiple myeloma	T/C	T/T	A/A	C/A	C/C	T/T
91	Male	69	multiple myeloma	C/C	T/T	A/A	C/C	C/C	T/T
92	Feale	65	multiple myeloma	C/C	C/T	A/A	C/A	C/C	C/C
93	Feale	61	multiple myeloma	T/C	T/T	A/A	C/C	C/C	T/T
94	Feale	61	multiple myeloma	T/C	C/T	A/A	C/C	C/C	T/T
95	Feale	57	multiple myeloma	C/C	T/T	A/A	C/C	C/C	C/T
96	Male	68	multiple myeloma	T/C	0/0	A/A	C/C	C/T	T/T
97	Male	43	multiple myeloma	C/C	T/T	G/A	C/A	C/C	C/T
98	Male	71	multiple myeloma	C/C	C/T	A/A	C/A	C/C	C/T
99	Male	66	multiple myeloma	T/C	C/T	G/A	C/A	C/T	T/T
100	Male	65	multiple myeloma	C/C	T/T	G/A	C/A	C/T	C/T
101	Male	51	multiple myeloma	C/C	T/T	A/A	C/C	C/C	T/T
102	Male	57	multiple myeloma	C/C	T/T	G/G	C/C	C/C	C/T
103	Feale	61	multiple myeloma	C/C	C/T	G/A	C/C	C/C	C/T
104	Feale	62	multiple myeloma	C/C	C/T	G/A	A/A	C/C	T/T
105	Male	52	multiple myeloma	T/C	T/T	G/A	A/A	C/T	C/T
106	Feale	48	multiple myeloma	C/C	T/T	G/A	C/A	C/T	T/T
107	Feale	53	multiple myeloma	T/T	T/T	A/A	C/C	C/C	T/T
108	Male	58	multiple myeloma	C/C	T/T	A/A	C/C	C/C	T/T
109	Male	49	multiple myeloma	C/C	T/T	G/A	C/C	0/0	C/T
110	Feale	61	multiple myeloma	T/C	T/T	G/A	0/0	C/C	T/T
111	Feale	62	multiple myeloma	T/T	C/T	A/A	C/C	C/C	T/T
112	Feale	50	multiple myeloma	T/C	T/T	G/A	C/A	C/C	C/T
113	Feale	64	multiple myeloma	C/C	C/T	A/A	C/A	C/C	T/T
114	Male	65	multiple myeloma	T/T	C/T	G/A	0/0	C/C	T/T
115	Male	55	multiple myeloma	C/C	T/T	G/A	A/A	C/C	C/T
116	Male	62	multiple myeloma	T/C	T/T	G/A	C/C	C/C	C/T
117	Feale	71	multiple myeloma	C/C	C/T	A/A	C/A	C/C	C/T
118	Male	52	multiple myeloma	C/C	T/T	G/A	C/A	C/C	T/T
119	Feale	57	multiple myeloma	T/C	C/T	A/A	C/C	C/T	T/T
120	Male	43	multiple myeloma	C/C	T/T	A/A	C/A	C/C	C/T
121	Male	63	multiple myeloma	T/T	0/0	A/A	C/C	C/C	C/T
122	Feale	64	multiple myeloma	C/C	C/T	G/A	C/C	C/C	T/T
123	Male	60	multiple myeloma	C/C	T/T	A/A	C/C	C/C	T/T
124	Male	67	multiple myeloma	C/C	C/T	A/A	C/A	C/C	T/T
125	Male	60	multiple myeloma	C/C	T/T	A/A	C/C	C/C	C/C
126	Feale	57	multiple myeloma	T/C	T/T	A/A	C/A	C/C	C/T
127	Feale	50	multiple myeloma	T/C	T/T	G/A	A/A	C/C	C/T
128	Male	48	multiple myeloma	T/T	T/T	A/A	C/A	0/0	0/0
129	Male	78	multiple myeloma	C/C	T/T	G/A	C/C	C/T	C/T
130	Feale	58	multiple myeloma	T/C	T/T	A/A	C/A	C/C	C/T
131	Male	64	multiple myeloma	C/C	T/T	A/A	C/C	C/C	C/T
132	Male	67	multiple myeloma	C/C	T/T	A/A	C/A	C/T	T/T
133	Male	48	multiple myeloma	C/C	T/T	A/A	C/A	C/T	T/T
134	Feale	47	multiple myeloma	T/C	T/T	A/A	C/A	C/C	C/C
135	Male	46	multiple myeloma	T/C	C/T	G/A	C/C	T/T	C/T
136	Male	68	multiple myeloma	T/C	T/T	G/A	C/C	C/T	T/T
137	Male	52	multiple myeloma	T/C	C/T	A/A	C/C	C/T	C/T
138	Male	66	multiple myeloma	T/C	T/T	A/A	C/A	C/C	C/C
139	Male	51	multiple myeloma	C/C	T/T	G/A	C/C	C/C	T/T
140	Feale	52	multiple myeloma	T/C	T/T	A/A	C/A	C/C	C/C
141	Feale	49	multiple myeloma	C/C	T/T	A/A	C/A	C/T	C/T
142	Feale	54	multiple myeloma	T/C	T/T	G/A	C/A	C/C	C/T
143	Male	57	multiple myeloma	T/T	0/0	0/0	C/A	0/0	C/T
144	Male	67	multiple myeloma	T/C	T/T	G/A	C/C	C/C	C/T
145	Feale	70	multiple myeloma	T/C	C/T	A/A	C/C	C/T	T/T
146	Male	58	multiple myeloma	T/C	C/T	G/A	C/A	C/C	C/T
147	Male	52	multiple myeloma	C/C	T/T	0/0	C/A	C/C	C/T
148	Feale	69	multiple myeloma	C/C	T/T	A/A	C/A	C/T	T/T
149	Feale	52	multiple myeloma	C/C	T/T	A/A	C/A	C/T	T/T
150	Male	65	multiple myeloma	C/C	T/T	A/A	C/C	T/T	C/C
151	Male	52	multiple myeloma	C/C	T/T	A/A	C/A	C/T	T/T
152	Male	68	multiple myeloma	T/C	T/T	A/A	C/C	C/C	T/T
153	Feale	64	multiple myeloma	C/C	C/T	A/A	C/C	C/C	T/T
154	Feale	59	multiple myeloma	T/C	C/T	A/A	C/A	C/C	T/T
155	Male	59	multiple myeloma	T/C	T/T	A/A	C/A	C/C	T/T
156	Male	55	multiple myeloma	C/C	T/T	G/A	C/C	C/C	C/C
157	Feale	62	multiple myeloma	C/C	T/T	A/A	C/A	C/C	C/T
158	Male	47	multiple myeloma	C/C	T/T	A/A	A/A	C/C	C/T
159	Male	53	multiple myeloma	C/C	T/T	G/A	C/A	C/C	C/T
160	Male	66	multiple myeloma	T/T	C/T	G/A	C/A	C/C	T/T
161	Feale	48	multiple myeloma	T/C	T/T	A/A	A/A	C/C	C/T
162	Male	51	multiple myeloma	C/C	T/T	A/A	C/C	C/C	T/T
163	Male	55	multiple myeloma	T/C	T/T	A/A	A/A	C/C	T/T
164	Male	64	multiple myeloma	C/C	T/T	A/A	C/C	C/C	C/T
165	Male	62	multiple myeloma	T/T	T/T	G/A	C/A	C/T	T/T
166	Feale	53	multiple myeloma	C/C	T/T	A/A	A/A	C/C	C/C
167	Feale	72	multiple myeloma	C/C	T/T	G/A	C/A	C/C	C/T
168	Feale	68	multiple myeloma	T/C	C/T	A/A	C/C	C/C	C/T
169	Feale	63	multiple myeloma	C/C	T/T	G/A	C/C	C/C	T/T
170	Feale	55	multiple myeloma	C/C	T/T	A/A	C/C	C/T	0/0
171	Feale	57	multiple myeloma	C/C	T/T	G/A	C/C	C/C	T/T
172	Feale	49	multiple myeloma	C/C	C/T	G/A	A/A	C/C	T/T
173	Male	58	multiple myeloma	T/C	T/T	G/A	C/C	C/C	C/C
174	Male	64	multiple myeloma	T/C	C/T	G/A	C/C	C/C	T/T
175	Feale	52	multiple myeloma	C/C	T/T	A/A	C/C	C/C	C/T
176	Feale	61	multiple myeloma	C/C	T/T	A/A	C/C	C/C	T/T
177	Male	59	multiple myeloma	T/C	C/T	G/A	C/A	C/C	C/T
178	Male	61	multiple myeloma	T/C	T/T	A/A	C/A	C/C	C/T
179	Feale	68	multiple myeloma	C/C	C/T	G/A	C/C	C/T	C/T
180	Male	54	multiple myeloma	C/C	T/T	A/A	C/C	C/T	T/T
181	Male	68	multiple myeloma	C/C	C/T	G/A	C/C	C/C	C/C
182	Male	80	multiple myeloma	C/C	T/T	A/A	C/A	C/C	C/T
183	Male	43	multiple myeloma	T/C	T/T	A/A	C/C	C/T	T/T
184	Male	54	multiple myeloma	C/C	T/T	A/A	C/A	C/C	C/T
185	Feale	57	multiple myeloma	C/C	T/T	A/A	C/A	C/C	T/T
186	Feale	49	multiple myeloma	C/C	C/T	G/A	A/A	C/C	T/T
187	Male	68	multiple myeloma	C/C	T/T	G/A	A/A	C/C	C/T
188	Male	56	multiple myeloma	C/C	T/T	G/A	C/C	C/C	T/T
189	Feale	50	multiple myeloma	C/C	C/T	G/A	C/A	C/C	T/T
190	Feale	58	multiple myeloma	C/C	T/T	A/A	C/C	C/C	T/T
191	Male	62	multiple myeloma	T/C	0/0	G/A	C/C	C/C	C/C
192	Male	51	multiple myeloma	C/C	T/T	A/A	C/A	C/C	T/C
193	Male	53	multiple myeloma	C/C	T/T	G/A	C/A	C/C	C/T
194	Male	70	multiple myeloma	C/C	T/T	G/A	C/C	C/C	T/T
195	Feale	52	multiple myeloma	C/C	T/T	A/A	C/A	C/C	T/T
196	Feale	69	multiple myeloma	T/C	0/0	G/A	A/A	C/C	C/T
197	Feale	54	multiple myeloma	T/C	T/T	A/A	C/A	C/C	T/T
198	Male	47	multiple myeloma	C/C	T/T	A/A	C/A	C/C	C/T
199	Male	48	multiple myeloma	T/T	C/T	A/A	C/A	C/C	C/T
200	Feale	66	multiple myeloma	T/C	T/T	A/A	C/C	C/C	T/T
201	Feale	53	multiple myeloma	C/C	T/T	A/A	C/C	C/C	T/T
202	Male	53	multiple myeloma	C/C	T/T	A/A	C/C	C/C	T/T
203	Feale	47	multiple myeloma	C/C	C/T	A/A	C/A	C/C	T/T
204	Male	60	multiple myeloma	C/C	T/T	A/A	C/C	C/C	C/T
205	Male	61	multiple myeloma	T/C	T/T	A/A	C/C	C/T	C/T
206	Male	58	multiple myeloma	T/C	T/T	G/A	C/C	C/C	T/T
207	Male	54	multiple myeloma	C/C	T/T	A/A	C/C	C/T	T/T
208	Male	51	multiple myeloma	C/C	T/T	A/A	C/C	C/C	C/T
209	Male	53	multiple myeloma	C/C	T/T	A/A	C/C	C/T	T/T
210	Male	79	multiple myeloma	C/C	T/T	A/A	A/A	C/C	T/T
211	Male	69	multiple myeloma	C/C	C/T	A/A	C/C	0/0	C/T
212	Feale	58	multiple myeloma	C/C	C/T	A/A	C/C	C/T	C/C
213	Feale	53	multiple myeloma	C/C	T/T	A/A	0/0	C/C	C/T
214	Feale	67	multiple myeloma	T/C	T/T	G/A	C/C	C/T	C/T
215	Male	59	multiple myeloma	T/C	T/T	A/A	C/A	C/C	T/T
216	Male	62	multiple myeloma	T/C	C/C	A/A	C/A	C/C	T/T
217	Male	65	multiple myeloma	C/C	T/T	A/A	C/C	C/T	T/T
218	Male	57	multiple myeloma	T/C	T/T	A/A	C/C	C/C	T/T
219	Male	73	multiple myeloma	C/C	T/T	A/A	C/C	C/C	C/T
220	Male	49	multiple myeloma	C/C	T/T	A/A	C/A	C/C	C/T
221	Male	43	multiple myeloma	C/C	T/T	A/A	C/A	C/C	C/T
222	Male	73	multiple myeloma	C/C	T/T	A/A	C/C	C/C	C/C
223	Feale	56	multiple myeloma	T/C	T/T	A/A	C/C	C/T	T/T
224	Feale	66	multiple myeloma	T/C	C/T	A/A	C/C	C/C	C/T
225	Male	58	multiple myeloma	C/C	T/T	A/A	C/A	C/C	C/T
226	Male	61	multiple myeloma	C/C	T/T	A/A	C/A	C/T	C/T
227	Feale	55	multiple myeloma	T/C	T/T	A/A	C/A	C/C	T/T
228	Male	53	multiple myeloma	T/C	T/T	A/A	C/C	C/T	T/T
229	Male	70	multiple myeloma	T/C	C/T	A/A	C/C	C/C	C/C
230	Feale	68	multiple myeloma	T/C	C/T	A/A	C/C	C/C	C/T
231	Feale	60	multiple myeloma	C/C	C/T	G/G	C/A	C/C	C/T
232	Male	61	multiple myeloma	C/C	T/T	A/A	C/C	C/C	C/C
233	Male	58	multiple myeloma	T/C	T/T	A/A	C/C	C/C	T/T
234	Male	60	multiple myeloma	C/C	T/T	A/A	C/A	C/C	T/T
235	Male	59	multiple myeloma	C/C	T/T	A/A	C/A	C/C	T/C
236	Male	78	multiple myeloma	T/C	T/T	A/A	C/A	C/C	T/T
237	Male	61	multiple myeloma	T/C	C/T	A/A	C/C	C/C	T/T
238	Feale	52	multiple myeloma	C/C	C/T	G/A	C/C	C/C	T/T
239	Male	61	multiple myeloma	C/C	T/T	A/A	C/A	C/C	C/T
240	Male	46	multiple myeloma	C/C	T/T	A/A	C/A	C/C	T/T
241	Male	56	multiple myeloma	C/C	T/T	G/A	C/C	C/C	T/T
242	Feale	53	multiple myeloma	C/C	T/T	A/A	C/A	C/C	T/T
243	Male	58	multiple myeloma	C/C	T/T	G/A	C/C	C/C	0/0
244	Male	56	multiple myeloma	C/C	C/T	G/A	C/A	C/C	C/T
245	Male	59	multiple myeloma	C/C	C/C	A/A	C/A	C/C	T/T
246	Male	70	multiple myeloma	C/C	T/T	A/A	C/A	C/C	T/T
247	Male	47	multiple myeloma	C/C	T/T	A/A	C/C	C/C	C/T
248	Male	58	multiple myeloma	T/C	T/T	G/A	C/C	C/C	T/T
249	Feale	50	multiple myeloma	C/C	T/T	G/A	C/A	C/T	T/T
250	Male	42	multiple myeloma	T/C	T/T	A/A	C/A	C/C	C/T
251	Feale	47	multiple myeloma	T/C	T/T	A/A	C/A	C/C	C/C
252	Male	67	multiple myeloma	T/C	0/0	A/A	C/A	C/C	C/T
253	Male	53	multiple myeloma	C/C	T/T	A/A	C/A	C/C	C/T
254	Feale	64	multiple myeloma	C/C	T/T	A/A	C/C	C/C	T/T
255	Male	57	multiple myeloma	T/C	T/T	A/A	C/A	C/C	C/T
256	Feale	49	multiple myeloma	T/C	T/T	A/A	C/A	C/C	T/T
257	Feale	57	multiple myeloma	T/C	C/T	A/A	C/C	C/T	T/T
258	Male	63	multiple myeloma	T/C	T/T	A/A	C/C	C/C	T/T
259	Male	45	multiple myeloma	T/T	T/T	A/A	C/C	C/C	T/T
260	Male	65	multiple myeloma	C/C	C/T	G/A	C/A	C/C	C/T
261	Male	85	multiple myeloma	C/C	T/T	A/A	C/A	C/C	C/T
262	Male	47	multiple myeloma	C/C	T/T	A/A	C/A	C/C	C/T
263	Feale	51	multiple myeloma	T/C	T/T	G/A	C/A	C/C	C/T
264	Feale	53	multiple myeloma	C/C	T/T	A/A	C/C	C/T	C/T
265	Male	52	multiple myeloma	C/C	C/T	G/A	C/C	C/C	C/T
266	Male	49	multiple myeloma	C/C	T/T	A/A	C/C	C/C	C/T
267	Male	63	multiple myeloma	T/C	T/T	A/A	C/C	C/C	T/T
268	Male	59	multiple myeloma	C/C	C/C	A/A	C/A	C/C	T/T
269	Male	53	multiple myeloma	T/T	T/T	G/G	C/C	C/C	T/T
270	Male	61	multiple myeloma	T/C	C/T	A/A	A/A	C/C	T/T
271	Feale	58	multiple myeloma	C/C	T/T	A/A	C/A	C/C	T/T
272	Male	51	multiple myeloma	T/C	C/T	G/A	C/C	C/C	T/T
273	Feale	65	multiple myeloma	C/C	T/T	G/A	C/C	C/C	C/C
274	Male	46	multiple myeloma	C/C	C/T	A/A	C/A	C/C	T/T
275	Male	61	multiple myeloma	T/C	T/T	A/A	C/A	C/C	C/C
276	Male	60	multiple myeloma	T/C	T/T	A/A	C/C	C/C	T/T
277	Male	58	multiple myeloma	C/C	T/T	A/A	C/A	C/C	T/T
278	Male	64	multiple myeloma	T/C	T/T	A/A	C/A	C/C	C/C
279	Feale	33	multiple myeloma	T/C	0/0	A/A	C/A	C/C	T/T
280	Feale	50	multiple myeloma	T/C	T/T	G/A	C/A	C/C	C/T
281	Feale	50	multiple myeloma	T/C	T/T	A/A	C/A	C/C	C/C
282	Male	66	multiple myeloma	C/C	C/T	A/A	C/C	C/C	C/C
283	Feale	44	multiple myeloma	T/C	T/T	A/A	A/A	C/T	C/T
284	Male	65	multiple myeloma	C/C	C/T	A/A	C/C	C/C	C/T
285	Male	52	multiple myeloma	T/T	T/T	A/A	C/C	C/C	T/T
286	Male	57	multiple myeloma	C/C	T/T	A/A	A/A	C/C	C/T
287	Male	45	multiple myeloma	T/T	T/T	G/A	C/C	C/C	T/T
288	Feale	67	multiple myeloma	T/C	T/T	A/A	C/C	C/T	T/T
289	Male	56	multiple myeloma	C/C	C/T	G/A	C/A	C/C	C/T
290	Male	53	multiple myeloma	T/C	T/T	G/A	C/A	C/C	C/T
291	Feale	70	multiple myeloma	T/C	C/C	A/A	C/C	C/C	C/C
292	Male	65	multiple myeloma	C/C	T/T	G/A	C/C	C/C	C/C
293	Male	66	multiple myeloma	C/C	T/T	G/A	C/A	C/C	C/T
294	Feale	62	multiple myeloma	T/C	T/T	G/A	C/C	C/C	T/T
295	Feale	56	multiple myeloma	T/T	C/T	A/A	C/C	C/C	C/T
296	Feale	59	multiple myeloma	T/C	C/T	G/G	C/A	C/T	C/T
297	Male	39	multiple myeloma	T/C	T/T	A/A	C/C	C/C	T/T
298	Feale	60	multiple myeloma	T/C	T/T	G/A	C/A	C/C	C/T
299	Feale	49	multiple myeloma	C/C	T/T	G/A	C/C	C/T	T/T
300	Male	68	multiple myeloma	T/T	0/0	A/A	C/A	C/C	T/T
301	Male	70	multiple myeloma	C/C	T/T	G/A	C/A	C/C	T/T
302	Male	62	multiple myeloma	T/C	C/T	A/A	A/A	C/C	C/C
303	Feale	42	multiple myeloma	C/C	T/T	A/A	C/C	C/C	C/T
304	Male	71	multiple myeloma	T/C	T/T	A/A	C/A	C/T	T/T
305	Male	50	multiple myeloma	T/C	T/T	G/A	C/A	C/T	C/T
306	Feale	50	multiple myeloma	C/C	T/T	A/A	C/C	C/T	C/T
307	Male	46	multiple myeloma	C/C	T/T	A/A	C/A	C/T	T/T
308	Feale	63	multiple myeloma	C/C	T/T	A/A	C/C	C/C	T/T
309	Male	64	multiple myeloma	C/C	T/T	A/A	C/C	C/C	C/T
310	Feale	52	multiple myeloma	C/C	C/T	G/A	C/C	C/C	T/T
311	Male	55	multiple myeloma	C/C	T/T	G/A	C/C	C/C	C/T
312	Feale	54	multiple myeloma	T/C	T/T	G/A	C/A	C/C	T/T
[illegible]	[illegible]	[illegible]	[illegible]	[illegible]	[illegible]	[illegible]	[illegible]	[illegible]	[illegible]
1392	Feale	58	healthy control	C/C	T/T	G/A	C/A	C/C	T/T
1393	Feale	59	healthy control	T/C	T/T	A/A	C/C	C/C	C/C
1394	Feale	42	healthy control	T/T	C/T	A/A	C/C	C/C	T/T
1395	Feale	63	healthy control	C/C	T/T	G/A	C/A	C/C	C/T
1396	Feale	58	healthy control	C/C	T/T	G/G	C/A	C/C	T/T
1397	Feale	24	healthy control	C/C	0/0	A/A	C/C	C/C	C/T
1398	Feale	48	healthy control	C/C	T/T	A/A	A/A	C/C	C/T
1399	Feale	56	healthy control	C/C	0/0	G/A	A/A	C/C	C/T
1400	Feale	44	healthy control	C/C	T/T	A/A	C/C	C/C	C/T
1401	Feale	34	healthy control	T/T	C/T	A/A	0/0	0/0	C/C
1402	Feale	41	healthy control	T/C	0/0	A/A	C/A	C/C	C/C
1403	Feale	60	healthy control	C/C	T/T	G/A	C/C	0/0	T/T
1404	Feale	42	healthy control	T/C	T/T	G/A	C/C	C/C	T/T
1405	Feale	43	healthy control	C/C	T/T	G/A	C/C	C/C	T/T
1406	Feale	45	healthy control	C/C	T/T	A/A	C/C	C/C	T/T
1407	Feale	46	healthy control	C/C	C/T	A/A	C/C	0/0	T/T
1408	Feale	49	healthy control	C/C	T/T	G/A	A/A	C/C	C/C
1409	Feale	53	healthy control	C/C	T/T	G/A	C/C	0/0	T/T
1410	Feale	55	healthy control	C/C	T/T	G/A	A/A	0/0	T/T
1411	Feale	51	healthy control	T/C	T/T	G/A	0/0	C/C	T/T
1412	Feale	59	healthy control	T/C	C/T	A/A	C/C	C/C	T/T
1413	Feale	57	healthy control	C/C	T/T	A/A	0/0	C/T	C/T
1414	Feale	57	healthy control	C/C	T/T	A/A	A/A	C/C	C/T
1415	Feale	58	healthy control	T/C	T/T	A/A	C/C	C/C	C/C
1416	Feale	58	healthy control	C/C	T/T	A/A	C/A	C/C	C/C
1417	Feale	59	healthy control	C/C	T/T	G/A	A/A	C/C	C/T
1418	Feale	59	healthy control	T/C	T/T	A/A	C/C	C/C	T/T
1419	Feale	60	healthy control	C/C	C/T	G/A	A/A	C/C	T/T
1420	Feale	60	healthy control	C/C	T/T	A/A	C/A	C/C	C/T
1421	Feale	60	healthy control	C/C	C/T	G/A	C/A	C/C	T/T
1422	Feale	61	healthy control	C/C	T/T	A/A	C/C	C/C	T/T
1423	Feale	61	healthy control	C/C	C/T	A/A	C/C	C/C	C/C
1424	Feale	62	healthy control	T/C	T/T	G/A	C/C	0/0	C/T
1425	Feale	68	healthy control	T/C	C/T	G/A	C/A	C/C	T/T
1426	Feale	69	healthy control	C/C	T/T	G/A	C/A	C/C	T/T
1427	Feale	69	healthy control	C/C	T/T	A/A	A/A	C/C	T/T
1428	Feale	48	healthy control	T/C	0/0	A/A	C/A	C/C	C/T
1429	Feale	62	healthy control	T/C	T/T	A/A	C/C	C/C	T/T
1430	Feale	48	healthy control	T/C	T/T	A/A	C/A	C/C	T/T
1431	Feale	39	healthy control	C/C	C/T	A/A	A/A	C/C	T/T
1432	Feale	54	healthy control	C/C	0/0	A/A	A/A	0/0	T/T
1433	Feale	40	healthy control	T/C	T/T	A/A	C/C	C/C	T/T
1434	Feale	46	healthy control	T/C	T/T	A/A	A/A	C/C	C/C
1435	Feale	71	healthy control	0/0	0/0	0/0	0/0	0/0	T/T
1436	Feale	47	healthy control	C/C	T/T	A/A	C/C	C/C	C/T
1437	Feale	48	healthy control	0/0	0/0	0/0	0/0	0/0	T/T
1438	Feale	37	healthy control	0/0	0/0	A/A	C/C	C/C	T/T
1439	Feale	51	healthy control	T/C	T/T	G/A	C/A	C/C	T/T
1440	Feale	59	healthy control	0/0	0/0	0/0	0/0	0/0	C/T
1441	Feale	42	healthy control	C/C	T/T	A/A	C/A	C/C	T/T
1442	Feale	62	healthy control	C/C	0/0	A/A	C/C	C/C	C/C
1443	Feale	44	healthy control	T/C	C/T	G/A	C/C	C/C	C/T
1444	Feale	51	healthy control	C/C	T/T	A/A	0/0	C/C	C/C
1445	Feale	39	healthy control	T/T	T/T	A/A	C/C	C/C	C/C
1446	Feale	39	healthy control	0/0	0/0	A/A	0/0	0/0	C/T
1447	Feale	48	healthy control	T/C	T/T	A/A	C/C	C/C	T/T
1448	Feale	56	healthy control	C/C	T/T	A/A	0/0	C/C	C/T
1449	Feale	26	healthy control	C/C	C/T	A/A	C/C	C/C	C/T
1450	Feale	41	healthy control	C/C	T/T	A/A	C/C	0/0	C/T
1451	Feale	43	healthy control	0/0	0/0	A/A	0/0	C/C	C/T
1452	Feale	59	healthy control	C/C	T/T	A/A	C/C	C/C	T/T
1453	Feale	63	healthy control	0/0	0/0	0/0	0/0	0/0	T/T
1454	Feale	40	healthy control	0/0	0/0	G/A	0/0	0/0	T/T
1455	Feale	62	healthy control	0/0	0/0	G/A	0/0	0/0	C/T
1456	Feale	44	healthy control	C/C	C/T	A/A	C/C	C/C	T/T
1457	Feale	46	healthy control	T/C	T/T	A/A	C/C	C/C	T/T
1458	Feale	54	healthy control	T/T	0/0	A/A	C/C	C/C	T/T
1459	Feale	49	healthy control	C/C	T/T	G/G	C/C	C/T	C/T
1460	Feale	45	healthy control	0/0	0/0	0/0	0/0	0/0	T/T
1461	Feale	44	healthy control	T/C	0/0	G/A	C/C	C/C	C/C
1462	Feale	39	healthy control	T/C	T/T	A/A	0/0	C/C	C/C
1463	Feale	42	healthy control	T/C	T/T	G/G	0/0	C/T	C/T
1464	Feale	46	healthy control	T/C	T/T	A/A	C/C	C/T	C/T
1465	Feale	50	healthy control	T/C	0/0	G/A	C/C	C/C	T/T
1466	Feale	52	healthy control	T/C	T/T	A/A	C/C	C/C	T/T
1467	Feale	52	healthy control	C/C	C/T	A/A	C/C	C/C	C/T
1468	Feale	42	healthy control	C/C	T/T	A/A	C/A	C/C	C/T
1469	Feale	54	healthy control	C/C	T/T	A/A	C/C	C/C	T/T
1470	Feale	38	healthy control	C/C	T/T	G/G	A/A	C/C	C/T
1471	Feale	77	healthy control	0/0	0/0	0/0	0/0	0/0	T/T
1472	Feale	50	healthy control	T/C	T/T	A/A	C/A	C/T	C/T
1473	Feale	43	healthy control	C/C	C/T	A/A	C/A	0/0	T/T
1474	Feale	44	healthy control	C/C	T/T	A/A	C/C	C/C	T/T
1475	Feale	41	healthy control	C/C	T/T	A/A	C/C	C/C	C/C
1476	Feale	42	healthy control	T/C	C/T	A/A	C/A	C/C	C/T
1477	Feale	29	healthy control	C/C	C/T	A/A	C/C	C/C	C/C
1478	Feale	38	healthy control	T/C	T/T	G/A	C/A	C/T	T/T
1479	Feale	38	healthy control	0/0	0/0	G/A	0/0	0/0	T/T
1480	Feale	50	healthy control	T/C	T/T	A/A	C/A	C/C	C/T
1481	Feale	37	healthy control	C/C	T/T	A/A	A/A	0/0	T/T
1482	Feale	40	healthy control	C/C	0/0	G/G	C/C	0/0	C/C
1483	Feale	43	healthy control	C/C	T/T	G/A	A/A	C/C	C/T
1484	Feale	39	healthy control	T/C	T/T	G/A	C/A	C/C	C/T
1485	Feale	49	healthy control	0/0	0/0	0/0	0/0	0/0	C/T
1486	Feale	47	healthy control	0/0	0/0	0/0	0/0	0/0	T/T
1487	Feale	47	healthy control	C/C	C/T	A/A	C/A	0/0	T/T
1488	Feale	47	healthy control	C/C	T/T	A/A	C/C	C/C	T/T
1489	Feale	41	healthy control	C/C	0/0	A/A	0/0	C/C	T/T
1490	Feale	45	healthy control	C/C	T/T	A/A	C/C	C/C	C/C
1491	Feale	41	healthy control	T/C	T/T	A/A	C/A	C/C	C/T
1492	Feale	42	healthy control	C/C	0/0	G/A	C/C	C/C	C/T
1493	Feale	39	healthy control	C/C	T/T	A/A	C/A	C/C	C/T
1494	Feale	42	healthy control	C/C	0/0	G/G	C/A	C/C	C/T
1495	Feale	45	healthy control	C/C	T/T	G/A	C/C	C/C	C/T
1496	Feale	37	healthy control	C/C	T/T	A/A	0/0	C/T	C/T
1497	Feale	45	healthy control	C/C	T/T	A/A	C/C	C/C	T/T
1498	Feale	57	healthy control	T/C	T/T	G/A	A/A	C/C	C/T
1499	Feale	56	healthy control	C/C	T/T	A/A	C/C	C/C	C/T
1500	Feale	74	healthy control	T/C	T/T	A/A	C/A	C/C	T/T
1501	Feale	58	healthy control	C/C	C/T	A/A	C/A	C/C	C/T
1502	Feale	60	healthy control	T/T	T/T	G/A	C/C	C/C	T/T
1503	Feale	52	healthy control	C/C	T/T	A/A	C/C	C/C	C/T
1504	Feale	54	healthy control	C/C	C/T	G/A	C/A	C/C	C/T
1505	Feale	64	healthy control	T/C	T/T	G/A	C/A	C/C	T/T
1506	Feale	60	healthy control	C/C	0/0	G/A	C/C	C/C	C/T
1507	Feale	72	healthy control	C/C	T/T	A/A	C/C	C/C	T/T
1508	Feale	50	healthy control	C/C	T/T	A/A	C/C	C/C	T/T
1509	Feale	55	healthy control	C/C	C/T	A/A	C/C	C/C	C/T
1510	Feale	57	healthy control	T/C	T/T	A/A	C/C	C/C	T/T
1511	Feale	62	healthy control	C/C	T/T	A/A	C/C	C/T	T/T
1512	Feale	60	healthy control	T/C	T/T	A/A	C/A	C/T	C/C
1513	Feale	45	healthy control	C/C	T/T	G/A	C/A	C/C	T/T
1514	Feale	52	healthy control	C/C	0/0	A/A	C/C	C/C	T/T
1515	Feale	61	healthy control	T/C	0/0	A/A	C/C	C/C	T/T
1516	Feale	52	healthy control	C/C	T/T	A/A	C/C	C/C	T/T
1517	Feale	36	healthy control	T/C	T/T	A/A	A/A	C/C	C/T
1518	Feale	61	healthy control	T/C	C/C	G/A	C/C	C/C	C/T
1519	Feale	59	healthy control	C/C	0/0	G/A	C/C	C/C	C/T
1520	Feale	52	healthy control	T/C	T/T	A/A	C/A	0/0	C/T
1521	Feale	68	healthy control	T/C	T/T	A/A	C/C	C/C	C/T
1522	Feale	59	healthy control	C/C	T/T	G/A	C/C	C/C	C/C
1523	Feale	60	healthy control	C/C	0/0	A/A	C/C	C/C	T/T
1524	Feale	48	healthy control	C/C	T/T	A/A	C/A	C/C	C/T
1525	Feale	62	healthy control	T/C	T/T	A/A	C/C	C/C	C/T
1526	Feale	66	healthy control	C/C	C/T	A/A	C/C	C/C	C/C
1527	Feale	70	healthy control	C/C	T/T	A/A	A/A	C/C	C/T
1528	Feale	69	healthy control	C/C	T/T	A/A	C/C	C/C	C/T
1529	Feale	65	healthy control	T/T	T/T	A/A	C/C	C/C	C/T
1530	Feale	66	healthy control	C/C	T/T	A/A	C/A	C/C	C/T
1531	Feale	73	healthy control	C/C	C/T	G/A	C/C	C/C	C/T
1532	Feale	66	healthy control	C/C	T/T	G/A	C/A	C/T	C/T
1533	Feale	79	healthy control	T/C	T/T	A/A	C/C	C/C	T/T
1534	Feale	66	healthy control	C/C	C/T	A/A	C/C	C/C	C/T
1535	Feale	73	healthy control	T/C	T/T	A/A	A/A	C/C	C/T
1536	Feale	61	healthy control	T/C	T/T	A/A	C/A	C/C	C/T
